# Protracted viral shedding and viral load are associated with ICU mortality in Covid-19 patients with acute respiratory failure

**DOI:** 10.1186/s13613-020-00783-4

**Published:** 2020-12-10

**Authors:** L. Bitker, F. Dhelft, L. Chauvelot, E. Frobert, L. Folliet, M. Mezidi, S. Trouillet-Assant, A. Belot, B. Lina, F. Wallet, J. C. Richard

**Affiliations:** 1 Service de Médecine Intensive Réanimation, Hôpital De La Croix Rousse, Hospices Civils de Lyon, 103 Grande Rue de la Croix Rousse, 69004 Lyon, France; 2 Université de Lyon, Université Claude Bernard, Lyon 1, INSA-Lyon, UJM-Saint Etienne, CNRS, Inserm, CREATIS, UMR 5220, U1206, 69621 Lyon, France; 3 CIRI, Centre International de Recherche en Infectiologie, Univ Lyon, Inserm, U1111, Université Claude Bernard Lyon 1, CNRS, UMR5308, ENS de Lyon, Team Virpath, 69007 Lyon, France; 4 Laboratoire de Virologie, Institut des Agents Infectieux, Hospices Civils de Lyon, National Reference Center for Respiratory Viruses, Department of Virology, Infective Agents Institute, North Hospital Network, Lyon, France; Virpath Laboratory, International Center of Research in Infectiology, INSERM U1111, CNRS-UMR 5308, École Normale Supérieure de Lyon, Université Claude Bernard Lyon, Université Claude Bernard, Lyon, France; 5 Université de Lyon, Université Claude Bernard, Lyon 1, Lyon, France; 6 Joint Research Unit Hospices Civils de Lyon-bioMérieux, Hospices Civils de Lyon, Lyon Sud Hospital, Pierre-Bénite, France; 7 National Referee Centre for Rheumatic AutoImmune and Systemic Diseases in childrEn (RAISE), Lyon, France; 8 Pediatric Nephrology, Rheumatology, Dermatology Unit, Hospices Civils de Lyon, Pierre-Bénite, France; 9 Service de Réanimation Polyvalente, Centre Hospitalier Lyon Sud, Hospices Civils de Lyon, Pierre-Bénite, France

**Keywords:** Acute respiratory failure, SARS-COV-2, COVID-19, Viral load, Viral shedding, Acute respiratory distress syndrome, Polymerase chain reaction

## Abstract

**Background:**

Protracted viral shedding is common in hospitalized patients with COVID-19 pneumonia, and up to 40% display signs of pulmonary fibrosis on computed tomography (CT) after hospital discharge. We hypothesized that COVID-19 patients with acute respiratory failure (ARF) who die in intensive care units (ICU) have a lower viral clearance in the respiratory tract than ICU patients discharged alive, and that protracted viral shedding in respiratory samples is associated with patterns of fibroproliferation on lung CT. We, therefore, conducted a retrospective observational study, in 2 ICU of Lyon university hospital.

**Results:**

129 patients were included in the study, of whom 44 (34%) died in ICU. 432 RT-PCR for SARS-CoV-2 were performed and 137 CT scans were analyzed. Viral load was significantly higher in patients deceased as compared to patients alive at ICU discharge (*p* < 0.001), after adjustment for the site of viral sampling and RT-PCR technique. The median time to SARS-CoV-2 negativation on RT-PCR was 19 days [CI_95 %_:15–21] in patients alive at ICU discharge and 26 days [CI_95 %_:17-infinity] in non-survivors at ICU discharge. Competitive risk regression identified patients who died in ICU and age as independent risk factors for longer time to SARS-CoV-2 negativation on RT-PCR, while antiviral treatment was independently associated with shorter time. None of the CT scores exploring fibroproliferation (i.e., bronchiectasis and reticulation scores) were significantly associated with time to SARS-CoV-2 negativation.

**Conclusions:**

Viral load in respiratory samples is significantly lower and viral shedding significantly shorter in ICU survivors of COVID-19 associated acute respiratory failure. Protracted viral shedding is unrelated to occurrence of fibrosis on lung CT.

## Background

COVID-19 infection is frequently associated with acute respiratory failure (ARF) with a median delay from symptom onset of 8–10 days [1, 2]. Approximately 14 to 28% of hospitalized patients require admission in intensive care units (ICU) [3–5]. ICU mortality is high, particularly in patients under invasive mechanical ventilation, and death is mainly related to multi-organ failure [6]. A dysregulated host-response leading to a cytokine storm has been suspected as potential mechanism involved in worsening respiratory failure and occurrence of shock [1, 7], and the optimal therapeutic regimen (antiviral, immunomodulatory or both) remains to be determined. However, protracted viral shedding is common in hospitalized patients with a median of 3 weeks [8], and may be longer in severe patients [9–12]. Whether ICU mortality is mainly related to uncontrolled viral infection, dysregulated host-response or both is currently unknown.

On the other hand, up to 40% of the patients with COVID-19 pneumonia display signs of pulmonary fibrosis on computed tomography (CT) after hospital discharge, and ICU admission has been identified as a potential risk factor [13]. Pulmonary fibrosis may be related to alveolar epithelial cell death related to viral infection, excessive immune response triggering pro-fibrotic response, and/or ventilator induced-lung injury.

We hypothesized that COVID-19 patients with ARF who die in ICU have a lower viral clearance in the respiratory tract than patients discharged alive, and that protracted viral excretion is associated with patterns of fibroproliferation on lung CT.

Hence, the primary objective of the study was to compare the time-to SARS-CoV-2 negativation assessed by real-time reverse transcriptase polymerase chain reaction (RT-PCR) in the group of patients alive at ICU discharge and in the group of patients who died in ICU. The secondary objective of the study was to evaluate the association of time-to SARS-CoV-2 negativation on RT-PCR with a CT-based score of fibroproliferation.

## Methods

### Study design

The study is a retrospective observational study, performed between February 2020 and June 2020, in 2 intensive care units of a university hospital (Hospices Civils de Lyon, France). This study was conducted in accordance with the amended Declaration of Helsinki and was approved by our institutional ethic committee (#20–41). Consent for data utilization was sought from the patients or their representative.

### Patients

Eligible participants were adults, presenting to the ICU with ARF related to COVID-19 pneumonia confirmed by a SARS-CoV-2 positive RT-PCR in respiratory swab during the 14 days preceding ICU admission or during the 7 days following ICU admission. Exclusion criteria were negative SARS-CoV-2 RT-PCR, previous inclusion in the same study during a prior ICU stay, and lack of consent to participate.

The ICU policy during the COVID-19 epidemic was to promote sequential RT-PCR sampling and chest CT every 7–10 days. Antiviral treatments deemed active against SARS-CoV-2 were provided only in case of inclusion in antiviral clinical trials. Steroids were not administered before negativation of RT-PCR as per ICU policies at the time the study was conducted.

### Data collection

The following variables were collected: demographic and anthropometric data, SAPS II [14] and SOFA scores [15] at ICU admission, immunosuppression status, fulfilment of acute respiratory distress syndrome (ARDS) criteria [16], date of first symptoms of COVID-19 infection, antiviral treatment active on SARS-CoV-2 at any time during ICU stay, respiratory and extra-respiratory organ support during ICU stay, ICU length of stay, date of chest CT performed during hospital stay and CT semi-quantitative scores (see below), date of sampling and results of each RT-PCR performed during hospital stay. Patients were considered immunocompromised if they had received chemotherapy within the last 6 months, uncontrolled HIV infection (CD4 T-cells less than 200 copies or 15%), corticosteroid therapy > 10 mg per day, immunosuppressive therapy within the last 3 months (6 months for Rituximab), aplasia, asplenia or splenectomy.

RT-PCR for SARS-CoV-2 were performed on nasopharyngeal swabs in non-intubated patients, and on nasopharyngeal swabs or lower respiratory tracts samples in patients under invasive mechanical ventilation. Due to evolution in routine diagnosis and diagnostic test shortage, RT-PCR were performed by 4 different techniques during the study period, targeting the ORF1a gene and with similar pre-analysis steps: 1-the QS technique developed by Institut Pasteur (Paris, France) was performed on 183 samples; 2; the QS technique adapted on the open access channel of the automated system Panther Fusion (Hologic ^®^, Marlborough, MA) was used on 180 samples (QS–HOL); 3- the assay Cobas^®^SARS-CoV-2 was used on the Cobas^®^ 6800 system (Roche Diagnostics ^®^, Bale, Switzerland) for 57 samples; 4- the Eurobioplex SARS-CoV-2 kit (Eurobio Scientific ^®^, Les Ulis, France) was used on 4 samples. For all techniques, RT-PCR results were expressed in terms of the cycle threshold value, which is defined as the number of cycles required for the signal to cross the threshold (i.e., exceed the background level). Cycle threshold values are inversely related to viral load, and samples were considered positive if the cycle threshold was ≤ 45. Cycle threshold values were lacking for 8 viral swabs performed in other hospitals using unknown RT-PCR technique. Time to SARS-CoV-2 negativation on RT-PCR was computed as the elapsed time from ICU admission to the time at which the first negative RT-PCR was reported, with no subsequent positive RT-PCR. Patients without SARS-CoV-2 negativation on RT-PCR during their hospital stay were right-censored at the time of their last positive RT-PCR.

All CT scans performed on patients of center#1 during their ICU stay were analyzed. CT acquisitions were performed in the supine position with an iCT 256 or Ingenuity CT (Philips Healthcare, Eindhoven, The Netherlands) using the following settings: voltage 120 to 140 kVP, slice thickness 0.9 mm to 1.5 mm, matrix size 512 × 512. Field of view, pixel size and mAs were adapted for each patient. Each CT scan were graded simultaneously by consensus by 2 pneumologists (LF, LC) using a semi-quantitative score [17]. Five CT slices were analyzed at the following location: aortic arch, 1 cm above the dome of the right hemidiaphragm, and on three additional levels equally spaced between these two levels. At each level, each lung was divided into an anterior and a posterior zone, creating four quadrants for analysis per level. The following radiologic patterns were assessed in each quadrant: reticulation, ground-glass opacification (GGO), and intense parenchymal opacification (IPO). Spatial extension of each pattern was graded using a semi-quantitative score: 0 = no involvement; 1 = < 5% involvement of lung area on the slice; 2 = 5% to 25% involvement; 3 = 26% to 49% involvement; 4 = 50% to 75% involvement; and 5 = > 75% involvement. For each radiologic pattern, the average score (range 0–20) was calculated by summing all quadrant values of all slices, divided by the number of slices. Finally, bronchiectasis was scored as present (1) or absent (0) on each slice, with the total bronchiectasis score calculated by summing values over all slices (range 0–5). Any fibroproliferation CT feature was defined for each patient as occurrence of at least one bronchiectasis score or reticulation score > 0 during ICU stay.

### Statistical analysis

The primary endpoint of the study was time to SARS-CoV-2 negativation on RT-PCR. The secondary endpoints of the study were maximal bronchiectasis, reticulation, GGO and IPO CT scores during ICU stay.

Statistical analyses were performed using R version 4.0.1 [18] and the following packages: survival [19, 20], survminer [21], cmprsk [22], powerSurvEpi [23], lme4 [24] and lmerTest [25]. A *p* value below 0.05 was chosen for statistical significance.

Categorical variables were expressed as count (percentage) and continuous data were expressed as medians (1^st^ quartile-3^rd^ quartile). Data were compared between groups with the Fisher’s exact test for categorical variables and the Mann–Whitney U test for continuous variables. 95% confidence intervals (CI_95 %_) for proportions were computed with the Wilson score method.

Evolution of viral load (assessed by the cycle threshold value) over time was analyzed using a linear mixed model, using time as a random slope and vital status at ICU discharge as a random intercept, and adjusting for the site of sampling (nasopharynx or lower respiratory tract) and RT-PCR technique. This adjustment was performed to take into account the potential confounding effect of site of sampling and RT-PCR technique on viral load.

The impact of ICU mortality and CT scores on time to SARS-CoV-2 negativation on RT-PCR were first analyzed without considering the competing risk of death using the Kaplan–Meier estimator and the log-rank test. Then, Fine and Gray competing risk regression analyses were performed to account for the competing risk of death on time to SARS-CoV-2 negativation on RT-PCR [26], and the proportional hazard assumption was assessed by graphical analysis of the Schoenfeld’s residuals. All multivariate analyses were performed by incorporating variables with *p* values ≤ 0.2 in univariate analysis and stepwise backward selection.

## Results

129 patients were included in the study (97 from center #1 and 32 from center #2). Patients’ characteristics are reported in Table 1. ICU mortality amounted to 34%.

**Table 1:** Patient’s characteristics at inclusion and during ICU stay

Variable	Overall population (*n* = 129)	Alive at ICU discharge (*n* = 85)	Deceased at ICU discharge (*n* = 44)	*p* value
Age (yr)	69 [59–77]	63 [58–73]	77 [68–82]	< 0.001
Sex male	94 (73%)	56 (66%)	38 (86%)	< 0.01
BMI (kg m^−2^)	28 [25–31]	28 [25–31]	28 [25–32]	NS
Immunosuppression	9 (7%)	4 (5%)	5 (11%)	NS
Time between 1st symptoms and ICU admission (day)	8 [6–11]	9 [6–12]	7 [5–10]	NS
Time between 1st RT-PCR and ICU admission (day)	1 [0–4]	1 [0–4]	1 [0–2]	NS
SAPS2	39 [32–52]	36 [28–46]	49 [39–53]	< 0.001
SOFA score at ICU admission	4 [2–7]	4 [2–6]	5 [3–8]	< 0.05
ARDS criteria	87 (67%)	51 (60%)	34 (81%)	< 0.05
Respiratory support				< 0.01
Oxygen or NIV or HFNO only	42 (33%)	35 (41%)	7 (17%)	
IV at any time	87 (67%)	50 (59%)	34 (83%)	
ECMO	6 (5%)	1 (1%)	5 (12%)	< 0.05
Vasopressor at any time	86 (67%)	50 (59%)	34 (81%)	< 0.05
RRT at any time	24 (19%)	13 (15%)	13 (30%)	NS
ICU LOS	16 [5–31]	17 [5–31]	15 [5–31]	NS
Antiviral treatment				
None	111 (86%)	73 (85%)	36 (86%)	NS
Immunoglobulin	1 (1%)	0 (0%)	1 (2%)	
Lopinavir-ritonavir	5 (4%)	4 (5%)	1 (2%)	
Lopinavir-ritonavir + IFN-β	2 (1%)	0 (0%)	2 (5%)	
Hydroxychloroquine	5 (4%)	3 (4%)	2 (5%)	
Remdesivir	5 (4%)	5 (6%)	0 (0%)	

Values are median [1st quartile-3rd quartile] or count (percentage)

*ARDS* acute respiratory distress syndrome, *BMI* body mass index, *ECMO* extracorporeal membrane oxygenation, *HFNO* high flow nasal oxygen, *ICU* intensive care unit, *IFN-β* interferon β, *IV* invasive ventilation, *LOS* length of stay, *NS* not significant, *RRT* renal replacement therapy, *RT-PCR* real-time reverse transcriptase polymerase chain reaction, *SAPS2* simplified acute physiology score

### Virologic tests

**Fig. 1:**
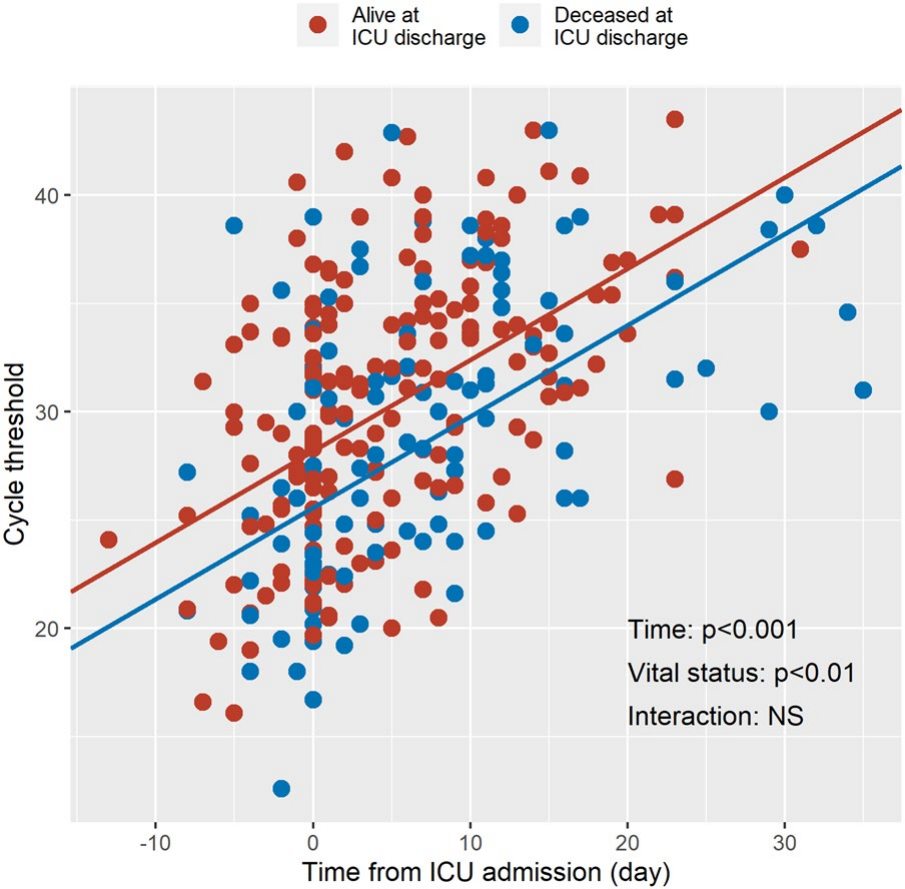
Viral load as a function of time from ICU admission in positive RT-PCR samples. Circles are individual RT-PCR measurements expressed as cycle threshold values (the lower the cycle threshold, the higher the viral load). Red and blue circles refer to patients alive and deceased at ICU discharge, respectively. Lines are regression lines for both groups, adjusted for the site of sampling (nasopharynx or lower respiratory tract) and RT-PCR technique. *ICU* intensive care unit, *RT-PCR* real-time reverse transcriptase polymerase chain reaction

432 RT-PCR for SARS-CoV-2 were performed, among which 121 (28%) were sampled from the lower respiratory tract, 311 (72%) from the nasopharynx, and 301 (70%) were positive. The median number of RT-PCR per patient amounted to 3 [2–4]. 40 patients (31%) had only 1 negative RT-PCR at end of follow-up, 35 (27%) patients had at least 2 consecutive negative RT-PCR at end of follow-up, and 54 (42%) still had a positive RT-PCR at end of follow-up. Timing of RT-PCR measurement for each patient is presented in Additional file 1. Cycle threshold values significantly increased over time from ICU admission (*p* < 0.001, Fig. 1 and Additional file 2), and were significantly lower (i.e., viral load was significantly higher) in patients deceased as compared to patients alive at ICU discharge, after adjustment for the site of sampling and RT-PCR technique. Interestingly, the effect of the site of sampling on viral load was not statistically significant (Additional file 2), with a regression coefficient amounting to 0.39 ± 0.82 (i.e., the fitted cycle threshold values were on average 0.39 ± 0.82 higher in nasopharynx samples that in lower respiratory tract samples, *p* = 0.63). A sensitivity analysis using only viral samples analyzed with the QS and the QS–HOL techniques gave identical results.

### Probability to change RT-PCR status

**Fig. 2:**
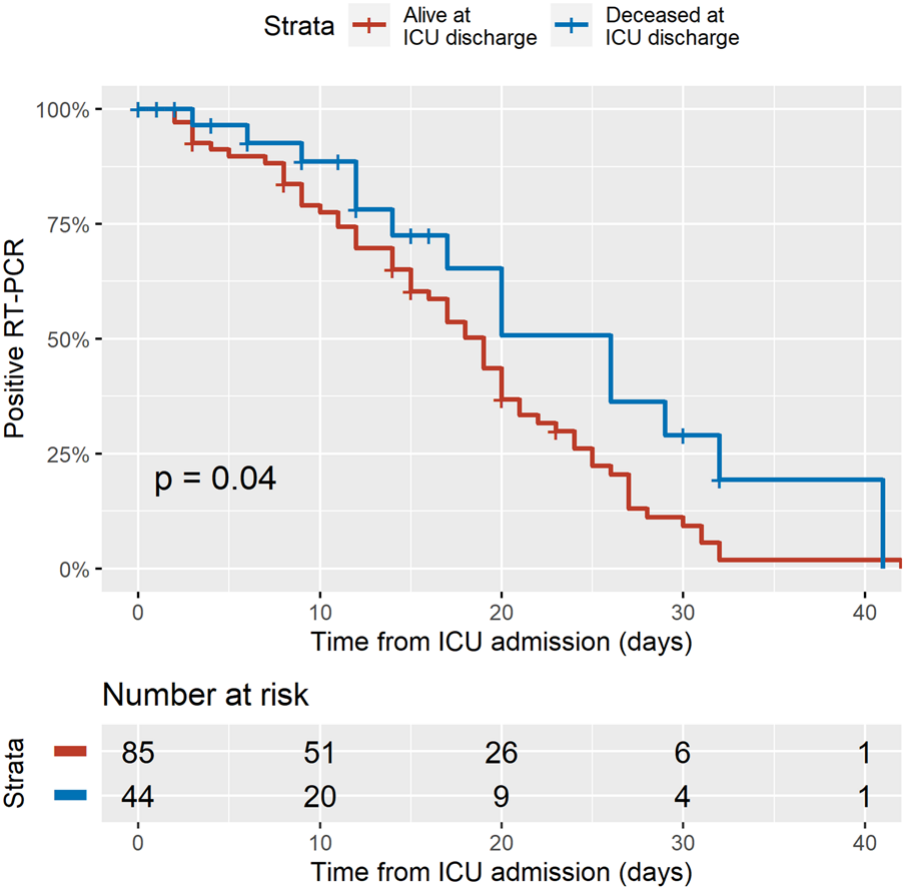
Kaplan–Meier plot of the probability of remaining RT-PCR positive for SARS-CoV-2. Blue line refers to patients deceased in ICU and red line to patients alive at ICU discharge. Right-censoring was performed at the time of the last positive RT-PCR in patients without SARS-CoV-2 negativation on RT-PCR during their hospital stay. *ICU* intensive care unit, *RT-PCR* real-time reverse transcriptase polymerase chain reaction

Kaplan–Meier representation of the probability of remaining positive to SARS-CoV-2 is presented in Fig. 2. Non-survivors at ICU discharge had a significantly longer viral excretion, as compared to ICU survivors (*p* < 0.05). The median time to SARS-CoV-2 negativation on RT-PCR was 19 days [CI_95 %_:15–21] in patients alive at ICU discharge, and 26 days [CI_95 %_:17-infinity] in non-survivors at ICU discharge.

**Fig. 3:**
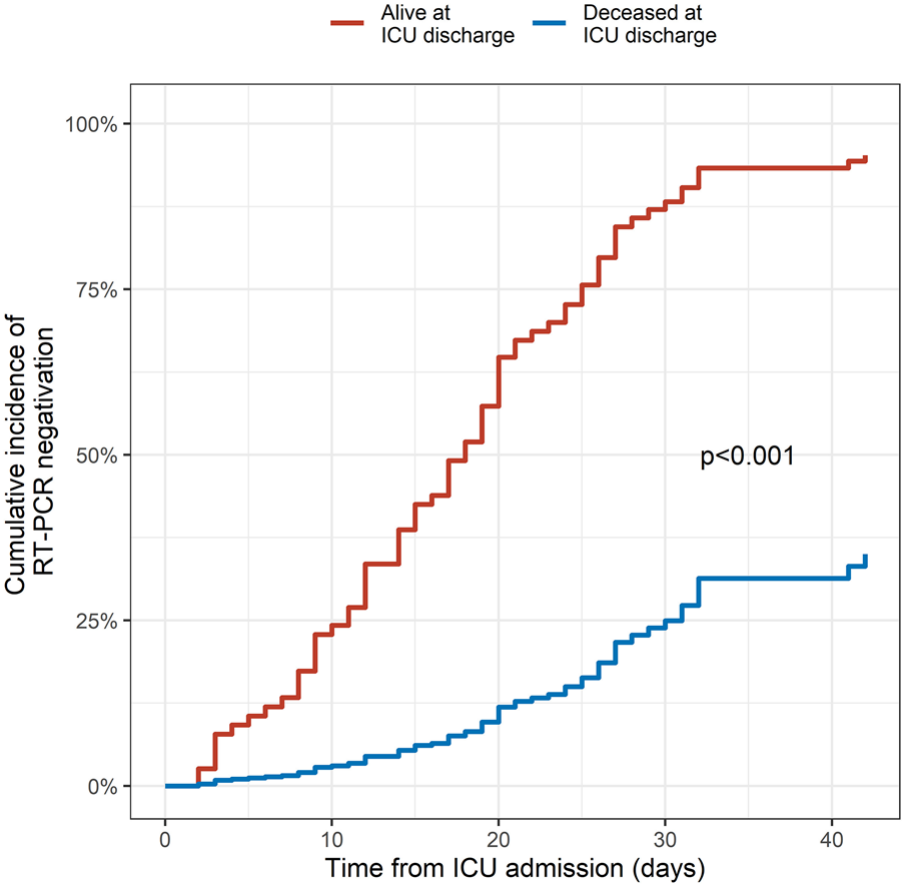
Multivariate fine and gray competitive risk regression. The model predicts the cumulative incidence of RT-PCR negativation following ICU admission. The curves represent multivariate model fit in patients deceased in ICU (blue lines) and in patients alive at ICU discharge (red lines). *ICU* intensive care unit, *RT-PCR* real-time reverse transcriptase polymerase chain reaction

Univariate and multivariate competitive risk regression of the probability of SARS-CoV-2 negativation on RT-PCR are presented in Table 2 and Fig. 3. Age and vital status at ICU discharge were independently associated with lower probability of SARS-CoV-2 negativation on RT-PCR, while antiviral treatment was independently associated with higher probability. Violation of the proportional hazard assumption was not identified for all tested variables. Sensitivity analyses using either a cycle threshold value ≤ 40 to define PCR positivity (Additional file 3) or time from 1^st^ positive RT-PCR instead of time from ICU admission (Additional file 4) provided identical results.

**Table 2:** Fine and gray competing risk regression of the probability of SARS-CoV-2 RT-PCR negativation

Variable	Univariate SHR [CI_95 %_]	Univariate *p* value	Multivariate SHR [CI_95 %_]	Multivariate *p* value
Vital status at ICU discharge * (ref = alive)	0.13 [0.07–0.25]	< 0.0001	0.15 [0.08–0.28]	< 0.0001
Age (per 1-year increment)	0.96 [0.94–0.98]	< 0.001	0.98 [0.96–0.99]	< 0.01
Antiviral treatment (ref = no)	2.19 [1.20–4.01]	< 0.05	2.38 [1.34–4.25]	< 0.01
Immunodeficiency (ref = no)	0.24 [0.06–0.96]	< 0.05	–	NS
SOFA at ICU admission (per 1-point increment)	0.95 [0.88–1.02]	0.16	–	NS
Sex (ref = male)	0.78 [0.49–1.22]	0.28	–	NS
Center (ref = center#1)	2.22 [1.45–3.40]	< 0.001	–	NS

*CI*_*95* *%*_ 95% confidence interval, *ICU* intensive care unit, *NS* non-significant, *ref* reference, *RT-PCR* real-time reverse transcriptase polymerase chain reaction, *SHR* subdistribution hazard ratio

### CT semi-quantitative scores

137 chest CT scans were performed in 39 patients of center #1 with at least one CT scan during their ICU stay (median number of CT scans per patient: 3 [2–4]).

The first CT and last CT scans for each patient were performed 2 [1–9] days and 28 [15–39] days after ICU admission, respectively (Additional file 5). Patients included in the CT sub-study had significantly higher SAPS2 and SOFA scores, met more frequently ARDS criteria, required more often organ support, and had significantly higher ICU mortality (Additional file 6). 22 patients (56% [CI_95 %_:41–71%]) exhibited patterns of fibroproliferation during their ICU stay (40% in the group of patients alive at ICU discharge and 74% in patients deceased in ICU, *p* = 0.05, Table 3). Maximal bronchiectasis and IPO CT scores during ICU stay were associated with ICU mortality, while maximal reticulation and GGO scores were not (Table 3).

**Table 3:** CT scores as a function of ICU mortality

Variables	Overall population (*n* = 39)	Alive at ICU discharge (*n* = 20)	Deceased at ICU discharge (*n* = 19)	*p* value
Maximal bronchiectasis score	1 [0–3]	0 [0–1]	2 [0–3]	< 0.05
Maximal reticulation score	0 [0–1]	0 [0–0]	0 [0–1]	0.15
Any fibroproliferation CT feature^a^	22 (56%)	8 (40%)	14 (74%)	0.05
Maximal GGO score	14 [11–15]	14 [13–15]	12 [9–15]	0.16
Maximal IPO score	9 [8–11]	8 [7–9]	11 [9–12]	< 0.01

Values are median [1st quartile-3rd quartile] or count (percentage)

*CT* computed tomography, *GGO* ground glass opacification, *ICU* intensive care unit, *IPO* intense parenchymal opacification

^a^Any fibroproliferation CT feature was defined as occurrence of at least one bronchiectasis score or reticulation score > 0 during ICU stay

**Fig. 4:**
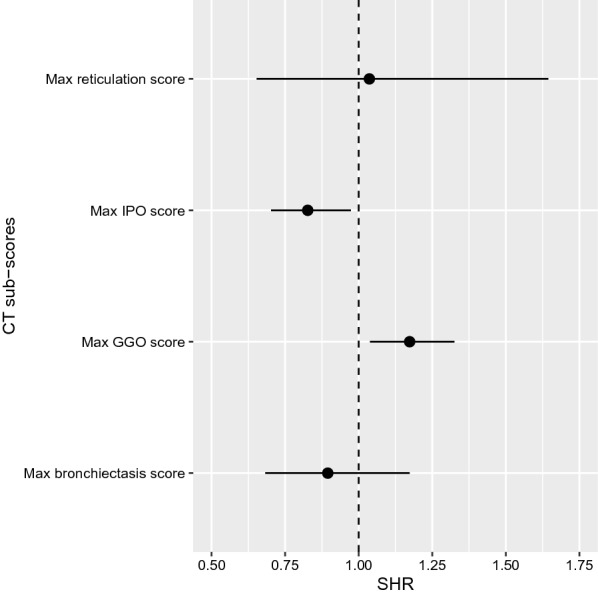
Fine and grey competitive risk regression of CT derived-scores to predict SARS-CoV-2 negativation on RT-PCR. The plot presents SHR estimates of CT-derived sub-scores to predict SARS-CoV-2 negativation on RT-PCR. Closed circles are SHR estimates and bars are their 95% confidence interval. Broken lines represent SHR equal to one. *CT* computed tomography, *GGO* ground glass opacification, *IPO* intense parenchymal opacification, *SHR* subdistribution hazard ratio, *RT-PCR* real-time reverse transcriptase polymerase chain reaction

Fine and Gray competing risk regression analysis of the relationship between the probability of SARS-CoV-2 negativation on RT-PCR and maximal CT scores during the ICU stay is presented in Fig. 4. The IPO score was associated with a lower probability of SARS-CoV-2 negativation on RT-PCR (i.e., the higher the score, the longer the time to negate RT-PCR), while the GGO score was associated with a higher probability of SARS-CoV-2 negativation on RT-PCR. None of the CT scores exploring fibroproliferation (i.e., bronchiectasis and reticulation scores) were significantly associated with time to SARS-CoV-2 negativation on RT-PCR. Multivariate analyses were not performed owing to the small number of patients in this analysis.

## Discussion

The main findings of the study are the following: 1- Time to SARS-CoV-2 negativation on RT-PCR is significantly longer and viral load significantly higher in respiratory samples of COVID-19 patients with ARF who died in ICU; 2- CT patterns of fibroproliferation are identified in more than 50% of the patients, but are not significantly associated with duration of viral shedding in respiratory samples; 3- The IPO CT score is associated with longer time to SARS-CoV-2 negativation on RT-PCR.

Regarding external validity, age, sex ratio in the present study were similar to previous ICU reports [1, 27–29]. Median time from symptoms to ICU admission was in the range of previously published studies on ICU patients [1, 27, 30]. Rate of patients requiring invasive ventilation, vasopressor administration and ICU mortality were similar to those observed in large ICU cohorts [28–30]. The relationship between duration of viral shedding and age has been previously observed in univariate analysis [8], and has been linked to immunosenescence. Demonstration of a lower duration of viral shedding in patients treated with antiviral agents is not unexpected as preliminary data suggest a positive effect on clinical outcome at least for remdesivir [31]. However, interpretation of the effect of antiviral treatments on viral shedding in the present study should be cautious as the number of patients is limited, the antiviral treatments were highly heterogeneous, and none of them has clearly shown any clinical efficacy apart remdesivir.

To our knowledge, no previously published study has related ICU mortality and duration of viral shedding in patients with ARF. Oppositely, a study on 191 hospitalized patients identified a significantly shorter viral shedding in non-survivors, but this study did not account for the competitive risk of death and mixed ICU and non ICU patients [11]. High viral load has been previously related to disease severity [12, 32], in line with the finding of the present study. It may be hypothesized that the longer duration of viral shedding in the non-survivors may be a consequence of the higher viral load at ICU admission. The longer viral shedding in non survivors may also be the consequence of ineffective immune response in this group, as our group has recently shown that up to 20% of ICU patients with COVID-19 and ARF presented with a sustained abrogation of type I interferon production [33], a major component of the innate immune system in the defense against viral infection. Taken together, these data suggest that efficient antiviral therapies are warranted to improve ICU mortality.

Several studies have analyzed chest CT with semi quantitative scores in non-severe patients with SARS-CoV-2 infection. In 21 repeatedly-studied patients from the early stage to the late phase of the disease, ground glass opacities were predominant 0–4 days after symptoms onset, crazy paving pattern was predominant 5–8 days after symptom onset, while the extent of IPO peaked at 10 days after symptoms onset [34]. Consistently with this finding, 100% of our patients exhibited IPO, as virtually all CT were performed more than 10 days after symptom onset. On 83 patients including 25 presenting with severe patients, total CT score and presence of IPO on CT were associated with disease severity [35]; a result in line with our results on more severely ill patients. In 3 studies, bronchus distortion was observed in 30% to 50% of the patients [36–38], a value similar to the 56% observed in the present study. We were unable to demonstrate an association between CT scores of fibroproliferation (reticulation and bronchiectasis scores) and protracted viral shedding, suggesting that lung fibrosis following COVID-19 may be related to host factors or external factors (such as ventilator-induced or patient self-inflicted lung injury) rather than to the intensity of viral aggression. However, the strong association between IPO score and duration of viral shedding suggests that patients with extensive IPO on CT are strong candidate for antiviral therapies. On the other hand, the association of GGO extent on CT and lower duration of viral shedding may suggest that GGO reflect recruitment of inflammatory cells in lung parenchyma, although this remains speculative.

Some limitations of the present study should be acknowledged. First, the estimated duration of viral shedding may have been overestimated by the relatively wide interval between RT-PCR samples (7–10 days), although this would be similar in both groups of patients (alive and deceased at ICU discharge). Second, we performed a majority of nasopharyngeal swabs for RT-PCR and this may have biased the results as false-negative RT-PCR may be less frequent in lower-respiratory-tract samples of ICU patients [39]. Third, 4 different RT-PCR techniques were used with expected impact on cycle threshold values, although this was accounted for by adjusting the multivariate analysis for the site of measurement and RT-PCR technique. Fourth, the cycle threshold defining RT-PCR positivity may be questionable, as the virus may be not cultivable anymore at such high values [40], and as positivity at this level might reflect the immune system deficiency and the impossibility to completely clear the virus. Nevertheless, the primary judgment criterion of the present study was unchanged using a lower cycle threshold (i.e., 40) in sensitivity analyses. Fifth, only 27% of the patients had at least 2 consecutive negative RT-PCR, and duration of viral shedding may have been underestimated by false negative measurements [8]. Finally, the CT score was computed in only 5 CT slices as previously performed in non-resolving ARDS [17], and a selection bias could not be ruled out, although the methodology was identical in all analyzed CT.

Nevertheless, the multicenter feature of the study and the relatively large sample size are important factors limiting the risk of bias. Although only a subset of patients could be analyzed by computed tomography, the number of CT scan analyzed ensured that the temporal time course of COVID-19 lung lesion has been correctly addressed. The demonstration of fibroproliferation on CT in the majority of the patients of the study suggest that anti-fibrotic therapies may be of interest to decrease duration of mechanical ventilation and prevent lung sequelae after ICU discharge.

## Conclusions

Viral load in respiratory samples is significantly lower and viral shedding significantly shorter in ICU survivors of COVID-19 associated respiratory failure, and is unrelated to occurrence of fibrosis on lung CT. Lung fibrosis is frequent in the most severe ICU patients, suggesting that anti-fibrotic therapies may be of interest to improve prognostic. The extension of intense parenchymal opacities on CT is associated with duration of viral shedding in respiratory samples suggesting that ICU patients presenting this pattern are strong candidate for potent antiviral therapies against SARS-CoV-2.

## Supplementary information

**Additional file 1.** Timing of rt-pcr sampling.

**Additional file 2.** Multivariate linear regression of variables associated with cycle threshold values (the inverse of viral load) in positive RT-PCR samples.

**Additional file 3.** Sensitivity analysis using a cycle threshold value ≤ 40 to define pcr positivity. multivariate fine and gray competitive risk regression of the probability of SARS-CoV-2 RT-PCR negativation.

**Additional file 4.** Sensitivity analysis using time from 1^st^ positive RT-PCR instead of time from ICU admission. Multivariate fine and gray competitive risk regression of the probability of SARS-CoV-2 RT-PCR negativation.

**Additional file 5.** Timing of chest computed tomography.

**Additional file 6.** Patient characteristics at ICU admission as a function of their inclusion in the chest ct sub-study.

## Data Availability

The datasets used and/or analyzed during the current study are available from the corresponding author on reasonable request.

## Abbreviations

ARDS: Acute respiratory distress syndrome
ARF: Acute respiratory failure
CI_95 %_: 95% confidence interval
CT: Computed tomography
GGO: Ground glass opacity
ICU: Intensive care unit
IPO: Intense parenchymal opacity
RT-PCR: Real-time reverse transcriptase polymerase chain reaction
