# Supplementary material for: Protracted viral shedding and viral load are associated with ICU mortality in Covid-19 patients with acute respiratory failure
Source: Ann Intensive Care. 2020 Dec 10;10:167. doi: 10.1186/s13613-020-00783-4 (PMC7725883; doi:10.1186/s13613-020-00783-4)
Supplement: Supplementary file 1 — Additional file 1. Timing of rt-pcr sampling. [file 13613_2020_783_MOESM1_ESM.docx]

**Title:** Protracted viral shedding and viral load are associated with ICU mortality in Covid-19 patients with acute respiratory failure: a two-center retrospective study

**Authors:** L BITKER, F DHELFT, L CHAUVELOT, E FROBERT, L FOLLIET, M MEZIDI, S TROUILLET-ASSANT, A BELOT, B LINA, F WALLET, JC RICHARD.

Additional file 1. Timing of RT-PCR sampling.


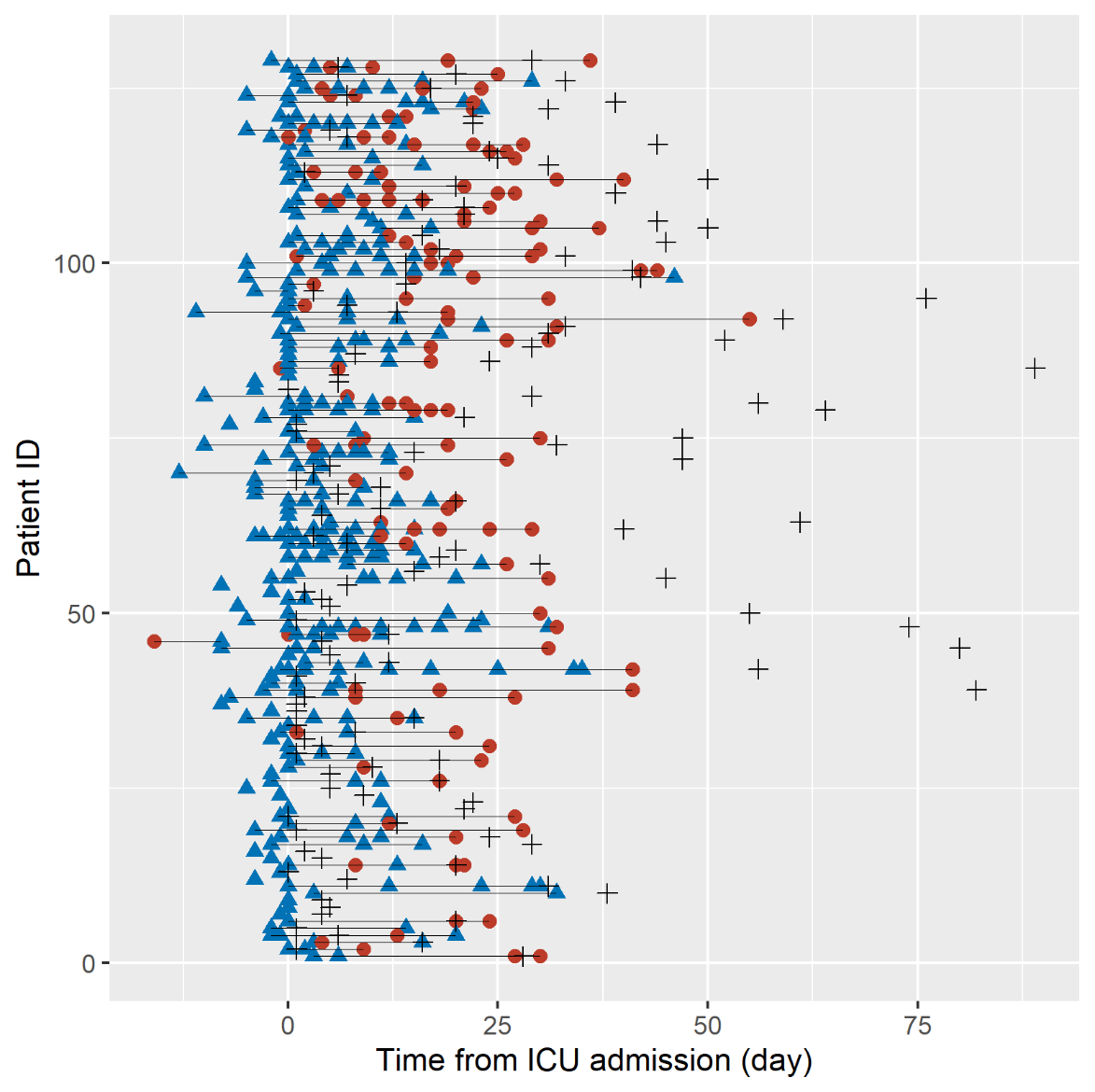


Each line refers to one patient, black crosses represent the time of ICU discharge, blue triangles represent positive RT-PCR, and red circles negative RT-PCR.

ICU = intensive care unit; RT-PCR = real-time reverse transcriptase polymerase chain reaction for SARS-CoV-2.
