# Supplementary material for: Protracted viral shedding and viral load are associated with ICU mortality in Covid-19 patients with acute respiratory failure
Source: Ann Intensive Care. 2020 Dec 10;10:167. doi: 10.1186/s13613-020-00783-4 (PMC7725883; doi:10.1186/s13613-020-00783-4)
Supplement: Supplementary file 2 — Additional file 2. Multivariate linear regression of variables associated with cycle threshold values (the inverse of viral load) in positive RT-PCR samples. [file 13613_2020_783_MOESM2_ESM.docx]

**Title:** Protracted viral shedding and viral load are associated with ICU mortality in Covid-19 patients with acute respiratory failure: a two-center retrospective study

**Authors:** L BITKER, F DHELFT, L CHAUVELOT, E FROBERT, L FOLLIET, M MEZIDI, S TROUILLET-ASSANT, A BELOT, B LINA, F WALLET, JC RICHARD.

Additional file 2. Multivariate linear regression of variables associated with cycle threshold values (the inverse of viral load) in positive RT-PCR samples.

| Variables | Model coefficient ± SE | Multivariate p value |
| --- | --- | --- |
| Intercept | 28.19 ± 1.11 | <0.001 |
| Time from ICU admission (per 1-day increment) | 0.42 ± 0.04 | <0.001 |
| Vital status at ICU discharge * (ref = alive) | -2.62 ± 0.84 | <0.01 |
| Site of sampling (ref = lower respiratory tract) | 0.39 ± 0.82 | 0.63 |
| RT-PCR technique (ref = QS)   - QS-HOL - ROCHE - EBX | 0.41 ± 0.72  -0.82 ± 1.07  1.12 ± 3.41 | 0.57  0.45  0.74 |

ICU =intensive care unit; ref = reference; RT-PCR = real-time reverse transcriptase polymerase chain reaction; SE = standard error.

QS = QS technique developed by Institut Pasteur (Paris, France); QS-HOL = QS technique adapted on the open access channel of the automated system Panther Fusion (Hologic ®, Marlborough, MA); ROCHE= assay cobas®SARS-CoV-2 used with the Cobas® 6800 system (Roche Diagnostics ®, Bale, Switzerland); EBX = Eurobioplex SARS-CoV-2 kit (Eurobio Scientific ®, Les Ulis, France).

Repetitive measurements per individual were taken into account by the use of a linear mixed model.
