# Supplementary material for: Protracted viral shedding and viral load are associated with ICU mortality in Covid-19 patients with acute respiratory failure
Source: Ann Intensive Care. 2020 Dec 10;10:167. doi: 10.1186/s13613-020-00783-4 (PMC7725883; doi:10.1186/s13613-020-00783-4)
Supplement: Supplementary file 3 — Additional file 3. Sensitivity analysis using a cycle threshold value ≤ 40 to define pcr positivity. multivariate fine and gray competitive risk regression of the probability of SARS-CoV-2 RT-PCR negativation. [file 13613_2020_783_MOESM3_ESM.docx]

**Title:** Protracted viral shedding and viral load are associated with ICU mortality in Covid-19 patients with acute respiratory failure: a two-center retrospective study

**Authors:** L BITKER, F DHELFT, L CHAUVELOT, E FROBERT, L FOLLIET, M MEZIDI, S TROUILLET-ASSANT, A BELOT, B LINA, F WALLET, JC RICHARD.

Additional file 3. Sensitivity analysis using a cycle threshold value ≤ 40 to define PCR positivity. Multivariate Fine and Gray competitive risk regression of the probability of SARS-CoV-2 RT-PCR negativation.


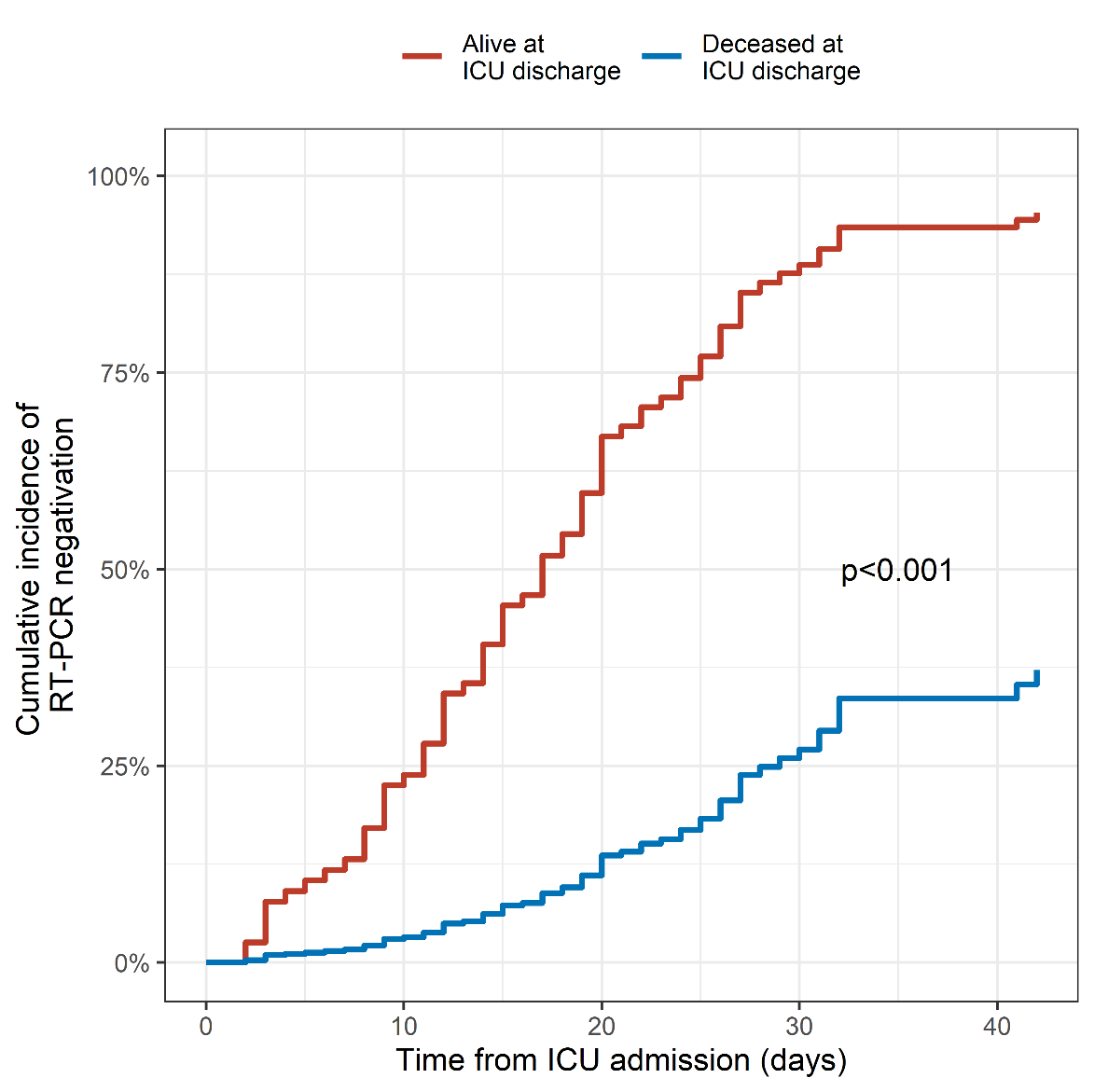


The curves represent multivariate model fit in patients deceased in ICU (blue lines) and in patients alive at ICU discharge (red lines).

ICU = intensive care unit; RT-PCR= real-time reverse transcriptase polymerase chain reaction.
