# Supplementary material for: Protracted viral shedding and viral load are associated with ICU mortality in Covid-19 patients with acute respiratory failure
Source: Ann Intensive Care. 2020 Dec 10;10:167. doi: 10.1186/s13613-020-00783-4 (PMC7725883; doi:10.1186/s13613-020-00783-4)
Supplement: Supplementary file 4 — Additional file 4. Sensitivity analysis using time from 1st positive RT-PCR instead of time from ICU admission. Multivariate fine and gray competitive risk regression of the probability of SARS-CoV-2 RT-PCR negativation. [file 13613_2020_783_MOESM4_ESM.docx]

**Title:** Protracted viral shedding and viral load are associated with ICU mortality in Covid-19 patients with acute respiratory failure: a two-center retrospective study

**Authors:** L BITKER, F DHELFT, L CHAUVELOT, E FROBERT, L FOLLIET, M MEZIDI, S TROUILLET-ASSANT, A BELOT, B LINA, F WALLET, JC RICHARD.

Additional file 4. Sensitivity analysis using time from 1^st^ positive RT-PCR instead of time from ICU admission. Multivariate Fine and Gray competitive risk regression of the probability of SARS-CoV-2 RT-PCR negativation.


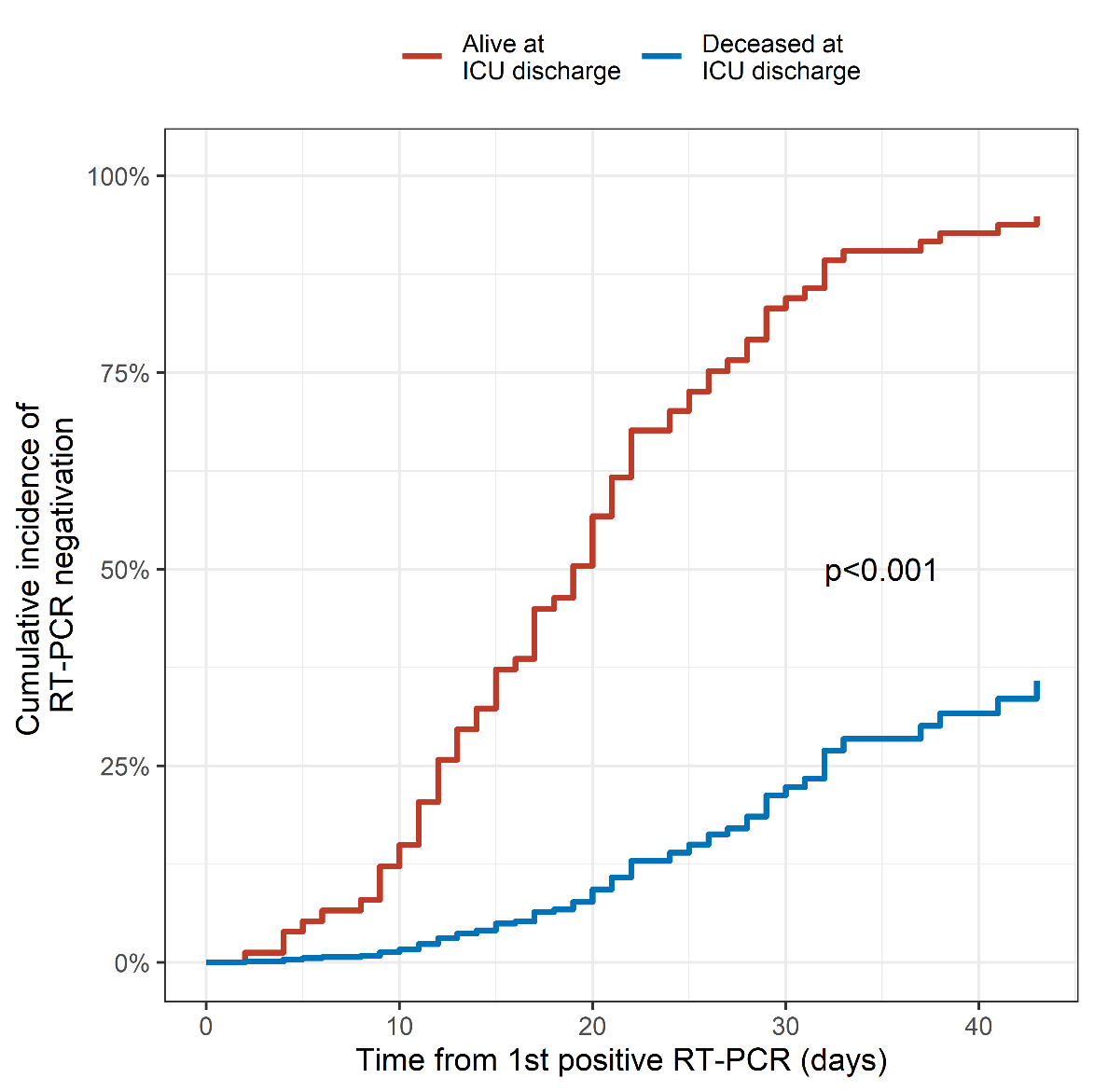


The curves represent multivariate model fit in patients deceased in ICU (blue lines) and in patients alive at ICU discharge (red lines).

ICU = intensive care unit; RT-PCR= real-time reverse transcriptase polymerase chain reaction.
