# Supplementary material for: Protracted viral shedding and viral load are associated with ICU mortality in Covid-19 patients with acute respiratory failure
Source: Ann Intensive Care. 2020 Dec 10;10:167. doi: 10.1186/s13613-020-00783-4 (PMC7725883; doi:10.1186/s13613-020-00783-4)
Supplement: Supplementary file 5 — Additional file 5. Timing of chest computed tomography. [file 13613_2020_783_MOESM5_ESM.docx]

**Title:** Protracted viral shedding and viral load are associated with ICU mortality in Covid-19 patients with acute respiratory failure: a two-center retrospective study

**Authors:** L BITKER, F DHELFT, L CHAUVELOT, E FROBERT, L FOLLIET, M MEZIDI, S TROUILLET-ASSANT, A BELOT, B LINA, F WALLET, JC RICHARD.

Additional file 5. Timing of chest computed tomography.


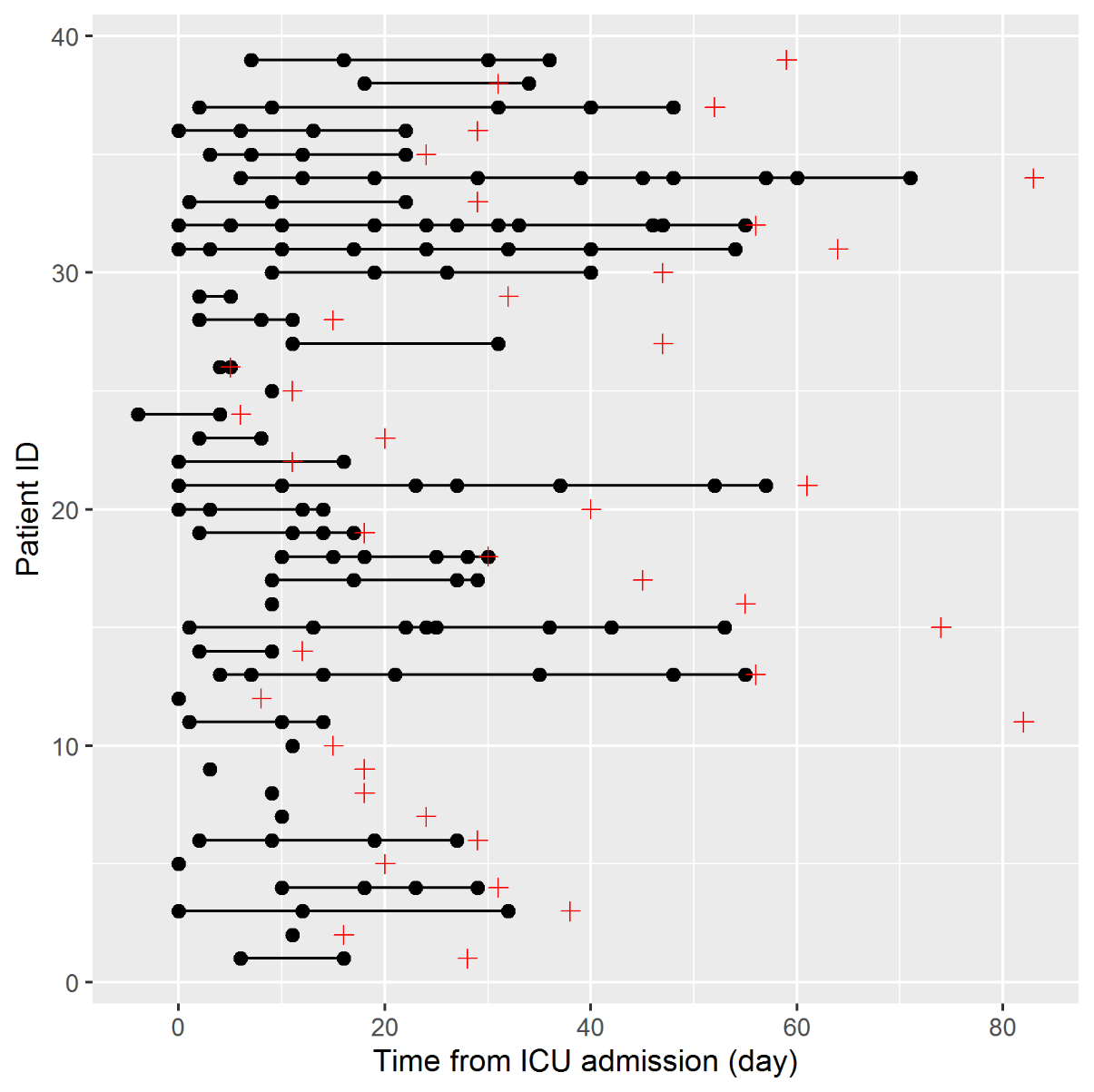


Each line refers to one patient, red crosses represent the time of ICU discharge and black circles represent timing of computed tomography relative to ICU admission.

ICU=intensive care unit.
