# Supplementary material for: Protracted viral shedding and viral load are associated with ICU mortality in Covid-19 patients with acute respiratory failure
Source: Ann Intensive Care. 2020 Dec 10;10:167. doi: 10.1186/s13613-020-00783-4 (PMC7725883; doi:10.1186/s13613-020-00783-4)
Supplement: Supplementary file 6 — Additional file 6. Patient characteristics at ICU admission as a function of their inclusion in the chest ct sub-study. [file 13613_2020_783_MOESM6_ESM.docx]

**Title:** Protracted viral shedding and viral load are associated with ICU mortality in Covid-19 patients with acute respiratory failure: a two-center retrospective study

**Authors:** L BITKER, F DHELFT, L CHAUVELOT, E FROBERT, L FOLLIET, M MEZIDI, S TROUILLET-ASSANT, A BELOT, B LINA, F WALLET, JC RICHARD.

Additional file 6. Patient characteristics at ICU admission as a function of their inclusion in the chest CT sub-study.

| Variables | Patient included in the CT sub-study  (n=39) | Patient not included in the CT sub-study  (n=90) | p value |
| --- | --- | --- | --- |
| Age (yr) | 67 [59-73] | 71 [60-78] | NS |
| Sex male | 30 (77%) | 64 (71%) | NS |
| BMI (kg.m^-2^) | 29 [26-33] | 28 [24-31] | NS |
| Time between 1^st^ symptoms and ICU admission (day) | 7 [6-11] | 9 [6-11] | NS |
| Time between 1^st^ RT-PCR and ICU admission (day) | 0 [0-2] | 1 [1-4] | <0.01 |
| SAPS2 | 46 [36-54] | 37 [30-48] | <0.05 |
| SOFA at ICU admission | 5 [4-8] | 3 [2-5] | <0.001 |
| ARDS criteria | 39 (100%) | 48 (53%) | <0.001 |
| Respiratory support   - Oxygen or NIV or HFNO only - invasive ventilation at any time | 0 (0%)  39 (100%) | 42 (47%)  48 (53%) | <0.001 |
| Vasopressor at any time | 38 (97%) | 48 (53%) | <0.001 |
| RRT at any time | 11 (28%) | 12 (13%) | <0.05 |
| ICU LOS | 29 [18-50] | 7 [3-21] | <0.0001 |
| Mortality | 19 (49%) | 25 (28%) | <0.05 |
| Antiviral treatment | 4 (10%) | 14 (16%) | NS |

Values are median [1^st^ quartile-3^rd^ quartile] or count (percentage).

ARDS = acute respiratory distress syndrome; BMI = body mass index; CT = computed tomography; HFNO = high flow nasal oxygen; ICU = intensive care unit; LOS = length of stay; NS = not significant; RRT = renal replacement therapy; RT-PCR= real-time reverse transcriptase polymerase chain reaction for SARS-CoV-2; SAPS2=simplified acute physiology score.
